# Responding to the COVID-19 Pandemic: A New Surgical Patient Flow Utilizing the Preoperative Evaluation Clinic

**DOI:** 10.1177/1062860620946741

**Published:** 2020-08-01

**Authors:** Sher-Lu Pai, Joan M. Irizarry-Alvarado, Nancy E. Pitruzzello, Wendelyn Bosch, Stephen Aniskevich

**Affiliations:** 1 Mayo Clinic, Jacksonville, FL

**Keywords:** coronavirus, perioperative surgical home, quality improvement, resumption of elective surgery, SARS-CoV-2, rRT-PCR testing

## Abstract

During the coronavirus disease 2019 (COVID-19) pandemic, the study institution recognized the importance of providing preoperative COVID-19 testing and symptom screening to ensure patient safety. A multidisciplinary quality improvement team used Define, Measure, Analyze, Improve, and Control methodology to understand the issues, identify solutions, and streamline patient flow. The existing preoperative evaluation (POE) clinic was utilized as a centralized entity to provide COVID-19 testing, symptom screening, and infection prevention education in addition to routine preoperative medical optimization. With the new process, the percentage of patients with COVID-19 testing results returned before surgery increased from 10% to 100%. Of the 593 asymptomatic patients screened by the POE clinic, 2 were found to have positive results. These patients had their surgeries postponed until proper recovery. The study institution has extended this new process to all surgical patients, warranting facility readiness for the resumption of elective surgery.

During the coronavirus disease 2019 (COVID-19) pandemic, the study institution recognized the importance of providing preoperative severe acute respiratory syndrome-coronavirus-2 (SARS-CoV-2) testing, symptom screening, exposure history documentation, and infection prevention education to ensure the safety of patients scheduled for essential surgical procedures. Patients who underwent surgeries during the incubation period could progress to respiratory failure or multi-organ dysfunction postoperatively, experiencing a higher morbidity and mortality rate.^1,2^ It had been shown that the incubation period for COVID-19 could persist for 14 days after exposure,^3^ leading to the possibility of presymptomatic transmission.^4,5^ Testing all asymptomatic surgical patients allowed physicians to make a clinical judgement on the timing of procedures. Testing also identified COVID-19 patients who would require specific facility setup and perioperative airway management to prevent transmission, thus minimizing exposure to health care providers.^6^

Symptom screening is another important aspect of risk stratification because there are no specific symptoms to distinguish COVID-19 from other respiratory infections.^7^ According to the Centers for Disease Control and Prevention (CDC) and many medical societies, screening should include an assessment of the following symptoms within the past 14 days: fever, headache, chills, new loss of taste or smell, cough, sore throat.^8,9^ Because patients might test negative during the asymptomatic incubation period, history regarding exposure to someone diagnosed with COVID-19 in the past 14 days also must be noted. Patients who were identified via symptom and exposure screening must be monitored appropriately for patient safety, contact tracing, and spread prevention.

## Methods

A multidisciplinary quality improvement (QI) team was formed, consisting of physicians, allied health staff members, and administrators from the departments of anesthesiology and perioperative medicine, general internal medicine, infectious disease, and surgery. DMAIC (define, measure, analyze, improve, control) methodology^10^ was used to understand the issues, identify possible solutions, and streamline a new surgical patient flow in response to the pandemic. This study was limited to analyzing scheduling and administrative data. None of the study team members had access to identifiable clinical records in connection with this project. Therefore, patient consent and institutional review board approval was not required per the study institution institutional review board guidelines.

### Define

On March 23, 2020, the SARS-CoV-2 ribonucleic acid (RNA) real-time reverse transcription–polymerase chain reaction (rRT-PCR) nasopharyngeal swab testing (Roche Molecular Diagnostics, Inc., Indianapolis, Indiana) became available at the institution. The Infection Prevention and Control (IPAC) and Surgical and Procedural Committee at the institution defined the goal to test all patients prior to their scheduled essential surgeries during the pandemic. The responsibility of ordering the test and screening for symptoms and exposure prior to surgeries was shared by the surgery and anesthesia teams. The plan also was to extend COVID-19 testing to all surgical patients when state and federal officials authorized the resumption of elective surgery.

### Measure

After excluding 33 emergent surgeries that did not allow time to await the results of the SARS-CoV-2 nasopharyngeal swab testing because of the patients’ critical conditions, 175 patients underwent essential surgeries from March 23, 2020, to March 29, 2020. Of the 175 patients, 18 (10%) patients had their testing results returned by the scheduled surgery start time.

### Analyze

**Figure 1.**
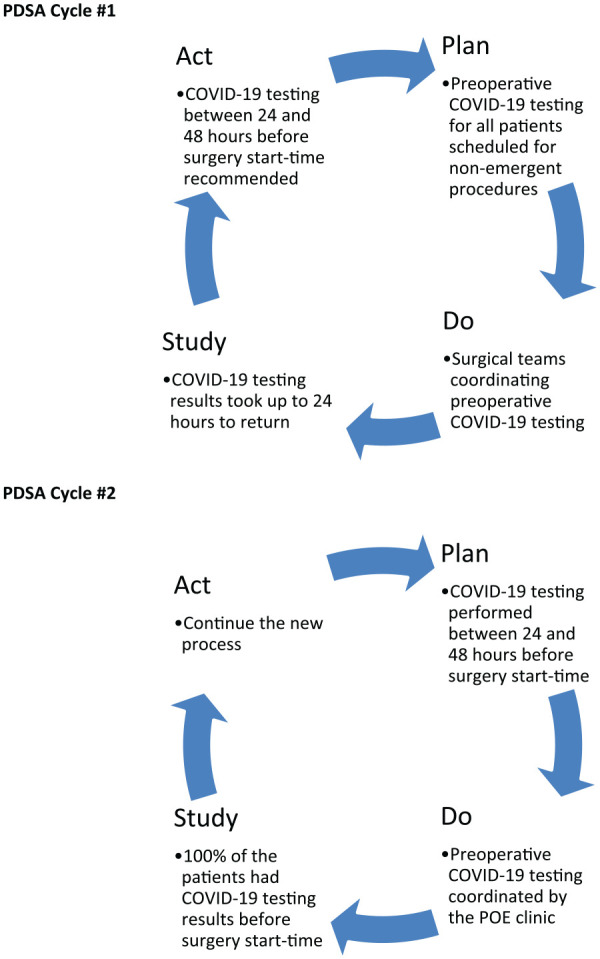
Plan-do-study-act cycles used to improve the percentage of patients with COVID-19 testing results before the scheduled surgery start time. Abbreviations: COVID-19, coronavirus disease 2019; PDSA, plan-do-study-act; POE, preoperative evaluation.

Although nonemergent surgical patients had received preoperative nasopharyngeal swab testing, only 10% of the results were returned by the scheduled surgery start time. Each surgical specialty team had a different preoperative COVID-19 testing and screening process, making it difficult to confirm the comprehensiveness and documentation of the symptom and exposure screening. The QI team also found that the SARS-CoV-2 RNA rRT-PCR test result could take up to 24 hours to be returned because of the governmental requirement on batch testing. On the other hand, the institutional IPAC recommended testing up to 48 hours prior to surgery followed by strict social isolation to minimize the possibility of community COVID-19 exposure. By using the Plan-Do-Study-Act methodology (Figure 1), the IPAC and QI team learned that the nasopharyngeal swab must be done between 24 to 48 hours prior to the start time to avoid delaying the surgery, yet still permitting a 24-hour time frame for patients to receive the swab testing via the designated drive-through testing site.

### Improve

The QI team determined the importance of finding a designated team to coordinate all COVID-19-related preoperative care and identified the preoperative evaluation (POE) clinic as the most impactful part of the patient flow to improve the process. For the last 7 years, the POE clinic has been providing preoperative testing, medical optimization, medication reconciliation, and patient education for more than 95% of the patients scheduled for nonemergent surgeries. The POE clinic team comprises anesthesiologists, internists, nurse practitioners, registered nurses, licensed practical nurses, and medical assistants. After a surgery is scheduled and a POE Assessment consult is ordered, the POE clinic’s registered or licensed practical nurses provide a triage phone call to each patient to confirm existing medical history and perform medication reconciliation. They may order additional testing following the guidelines established by the clinic physicians. Outside medical records, specifically cardiac testing, are often requested for the 3 years prior to the POE clinic appointment. Patients are scheduled to be seen at the POE clinic by a physician or a nurse practitioner, depending on the number of preexisting comorbidities.^11^ All medical conditions, preexisting or newly diagnosed, are optimized prior to the surgery. During the POE clinic visit, patients receive preparatory education on individualized perioperative medication instructions, fasting guidelines, opioid alternatives for perioperative pain control, and specific informational materials designed for different surgery and anesthetic modalities. The complete evaluation from the POE clinic serves as the admission history and physical documentations for these surgical patients.

On March 30, 2020, the QI team implemented a new process utilizing the POE clinic to provide SARS-CoV-2 testing, symptom screening, patient education, and preoperative optimization for patients scheduled for essential surgeries. During POE triage phone calls, the nurses provided education on strict social isolation, personal hygiene, and face mask usage in addition to the routine review of medical history and medication reconciliation. Patients were advised to follow these recommendations, especially after nasopharyngeal swab testing. Because some patients might require tending by their caregivers postoperatively, the decision to test 1 designated, non-interchangeable caregiver for each patient was made by physicians on a case-by-case basis.

Patients with low viral replication of COVID-19 in the upper respiratory tract could test negative for the SARS-CoV-2 RNA rRT-PCR nasopharyngeal swab results. If clinical signs or symptoms suggestive of COVID-19 infection were identified by symptom screening in patients with negative results, these patients were referred to the COVID-19 Virtual Clinic (CVC) for further evaluation. Here a chest X-ray, electrocardiography, and laboratory tests might be necessary to determine if there were any clinical indications of COVID-19 infection or another disease process. The POE clinic served as an important centralized location to alert surgical staff to potentially infected patients before they arrive at the institution for evaluation, thus avoiding further contagion.

If SARS-CoV-2 RNA was detected, patients were immediately referred to the CVC for further telephone or video follow-up, and the surgeries were postponed. CVC physicians would provide instructions on self-isolation, mitigation of infection spread, and, ultimately, retesting according to CDC guidelines.^12^ If SARS-CoV-2 RNA was not detected, patients were seen at the POE clinic following the routine preoperative history and physical examination process already in place prior to the pandemic. During this preoperative visit, additional screening for symptoms of COVID-19, recent travel history, and contact history with symptomatic or COVID-19 positive persons were inquired about to further assess the risk of infection before the patient proceeded with surgery. Other testing, such as a chest X-ray, might be ordered per the physician’s clinical judgement. Education on the prevention of COVID-19 infection and spread was reinforced again during the visit.

### Control

The POE clinic and operating room schedules were reviewed weekly to evaluate the effectiveness of the new patient flow and to determine whether changes to the process were needed.

## Results

**Figure 2.**
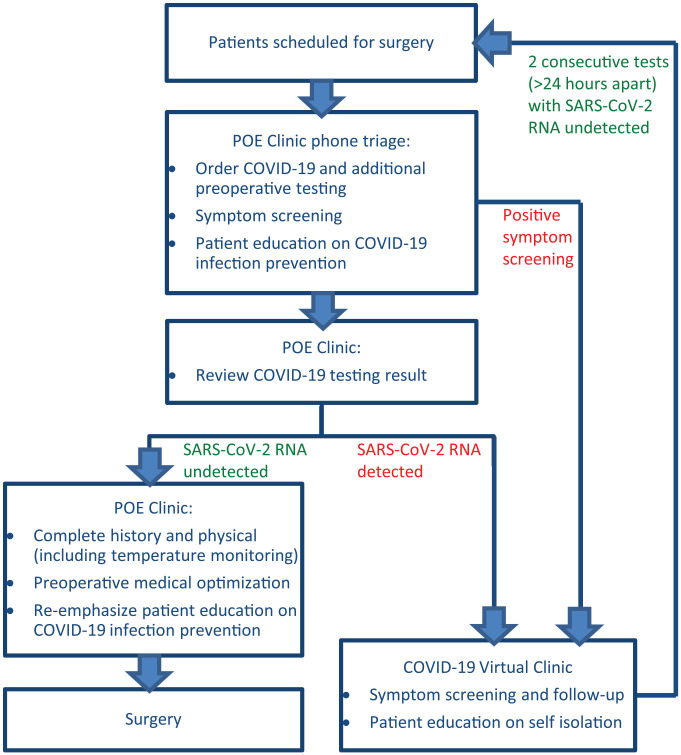
New preoperative evaluation clinic patient flow in response to the COVID-19 pandemic. Abbreviations: COVID-19, coronavirus disease 2019; POE, preoperative evaluation; RNA, ribonucleic acid; SARS-CoV-2, severe acute respiratory syndrome-coronavirus-2.

From March 31, 2020, to April 24, 2020, a total 593 patients successfully underwent the preoperative SARS-CoV-2 RNA testing, symptom screening, patient education, and preoperative optimization for their scheduled surgeries following the new process (Figure 2). Of these patients, 2 were found to be positive from the preoperative testing. They did not proceed with their scheduled surgeries and were referred to the CVC. All 593 (100%) of the patients had their testing results before the scheduled surgery start time; thus, surgeries were not delayed because of lab processing. For emergent life- or limb-threatening surgeries for which physicians felt that a delay caused by waiting for the SARS-CoV-2 RNA results would adversely affect the patients’ well-being, all perioperative staff members would proceed with the surgeries as if the patients had tested positive for SARS-CoV-2.

## Discussion

Robust COVID-19 screening and testing is one of the strategies health care facilities may implement in response to the pandemic, in addition to universal masking, enhanced cleaning, and contact tracing. The success of the new process is related to the availability of SARS-CoV-2 RNA tests. If facilities do not have enough tests available, resuming elective surgery may need to be delayed until testing ability improves and evidence-based infection prevention techniques are in place.^13^ It is beyond the scope of this article to discuss the exact sensitivity and specificity of SARS-CoV-2 RNA rRT-PCR testing. As testing capacity and quality of sample methodology continues to increase, testing of multiple specimens from oropharynx, sputum, feces, or other sites may improve the sensitivity and reduce false-negative test results.^14^ However, a rRT-PCR nasopharyngeal swab is currently considered the gold standard for testing patients with acute illness.^15^ As the COVID-19 rapid antibody serology test becomes available, another new testing workflow may be established in the near future in order to determine convalescent state. To improve and expedite turnaround time of preoperative COVID-19 batched testing, an institution can consider a separate testing location for nonexposure and asymptomatic patients scheduled for nonemergent procedures. This would decrease patient wait time, scheduling issues, and improve coordination of COVID-19 screening for this patient population.

The new process map also is designed under the assumption that sufficient personal protective equipment is available for all perioperative staff members. With intubation and extubation being high-risk aerosol-generating procedures and the possibility of a false negative SARS-CoV-2 RNA result, an N95 mask and a full face shield are still required for all anesthesiologists, nurse anesthetists, and other staff members during airway management for patients who tested negative. One of the patients initially tested negative during the preoperative testing process, but SARS-CoV-2 RNA was detected in the nasopharynx during this patient’s postoperative hospital stay. When staff exposure to COVID-19 was suspected because of the new diagnosis, the institution identified staff members via contact tracing and provided testing to all involved.

Little is known about when to allow a patient who has recovered from COVID-19 to undergo surgery safely. For patients with SARS-CoV-2 RNA detected via nasopharyngeal swabs, the exact viral shedding and infectivity of these patients are still under investigation, whereas the rRT-PCR test result does not distinguish between infectious virus and noninfectious RNA.^16^ The study team’s belief is that these patients should be delayed until completely afebrile, asymptomatic from a respiratory standpoint, and have at least 2 consecutive negative COVID-19 results more than 24 hours apart.^12^ Until more is known about transmissibility, institutions may wish to consider these patients to be COVID-19 positive if surgery is needed in close proximity to their illness.

In response to the COVID-19 pandemic, physicians and health care organizations had followed recommendations from the CDC, state and federal government agencies, and medical specialty societies to cancel nonessential surgical procedures.^17-19^ In anticipation of the end of the pandemic, several specialty societies released statements and recommendations for resuming elective surgery.^6,13^ When state and federal officials authorize the resumption of these surgeries, hospitals, surgical centers, and offices must keep the safety of patients and surgical staff members a priority. Strategic planning regarding testing, symptom screening, and patient education may improve facility readiness to safely resume elective surgery. Using existing infrastructures similar to the POE clinic framework, hospitals, surgical centers, and offices may create similar processes to improve facility readiness for resuming elective surgery. The POE clinic plans to extend this process to all surgical patients once the institution resumes care of patients who postponed their elective surgical procedures during the pandemic. The POE clinic also intends to expand the number of virtual POE clinic appointments for patients with no major comorbidities. The virtual visits would still confirm the patient’s existing medical history and provide patient education as in the in-person visits, but the physical examination would be completed in the preoperative holding areas by anesthesiologists on the day of the surgery. With a centralized entity to coordinate COVID-19 preoperative testing, result follow-up, symptom screening, and patient education in addition to proper medical optimization, the streamlined process may ensure workflow efficiency and patient safety.
